# Lessons for TB from the COVID-19 response: qualitative data from Brazil, India and South Africa

**DOI:** 10.5588/pha.23.0044

**Published:** 2023-12-07

**Authors:** H. Myburgh, M. Kaur, P. Kaur, V. Santos, C. Almeida, G. Hoddinott, D. T. Wademan, P. V. M. Lakshmi, M. Osman, S-A. Meehan, A. C. Hesseling, A. Purty, U. B. Singh, A. Trajman

**Affiliations:** 1 Desmond Tutu TB Centre, Department of Paediatrics and Child Health, Faculty of Medicine and Health Sciences, Stellenbosch University, Cape Town, South Africa; 2 Amsterdam Institute for Social Science Research (AISSR), University of Amsterdam, Amsterdam; 3 Amsterdam Institute for Global Health & Development Amsterdam, The Netherlands; 4 Department of Community Medicine & School of Public Health, Post Graduate Institute of Medical Education and Research, Chandigarh, India; 5 Prefeitura Municipal De Quissamã: Health, Rio de Janeiro, RJ; 6 Department of Internal Medicine, Medical School, Federal University of Rio de Janeiro, Rio de Janeiro, RJ, Brazil; 7 School of Human Sciences, Faculty of Education, Health & Human Sciences, University of Greenwich, London, UK; 8 Pondicherry Institute of Medical Sciences, Department of Community Medicine, Puducherry; 9 All India Institute of Medical Sciences, Department of Microbiology, New Delhi, India; 10 Department of Internal Medicine, Medical School, Federal University of Rio de Janeiro, Rio de Janeiro, RJ, Brazil; 11 McGill University, Montreal, QC, Canada

**Keywords:** TB, COVID-19, community engagement, political will, recovery

## Abstract

**BACKGROUND:**

Brazil, India and South Africa are among the top 30 high TB burden countries globally and experienced high rates of SARS-CoV-2 infection and mortality. The COVID-19 response in each country was unprecedented and complex, informed by distinct political, economic, social and health systems contexts. While COVID-19 responses have set back TB control efforts, they also hold lessons to inform future TB programming and services.

**METHODS:**

This was a qualitative exploratory study involving interviews with TB stakeholders (*n* = 76) in Brazil, India and South Africa 2 years into the COVID-19 pandemic. Interview transcripts were analysed using an inductive coding strategy.

**RESULTS:**

Political will – whether national or subnational – enabled implementation of widespread prevention measures during the COVID-19 response in each country and stimulated mobile and telehealth service delivery innovations. Participants in all three countries emphasised the importance of mobilising and engaging communities in public health responses and noted limited health education and information as barriers to implementing TB control efforts at the community level.

**CONCLUSIONS:**

Building political will and social mobilisation must become more central to TB programming. COVID-19 has shown this is possible. A similar level of investment and collaborative effort, if not greater, as that seen during the COVID-19 pandemic is needed for TB through multi-sectoral partnerships.

## Abstract (fr)

**CONTEXTE:**

Le Brésil, l’Inde et l’Afrique du Sud figurent parmi les 30 pays les plus touchés par la TB dans le monde et ont connu des taux élevés d’infection et de mortalité dus au SARS-CoV-2. La réponse au COVID-19 dans chacun de ces pays a été sans précédent et complexe, en raison de contextes politiques, économiques, sociaux et de systèmes de santé distincts. Si les réponses au COVID-19 ont fait reculer les efforts de lutte contre la TB, elles permettent également de tirer des enseignements pour les futurs programmes et services de lutte contre la TB.

**MÉTHODES:**

Il s’agit d’une étude exploratoire qualitative comprenant des entretiens avec des acteurs de la lutte contre la TB (*n* = 76) au Brésil, en Inde et en Afrique du Sud, 2 ans après le début de la pandémie de COVID-19. Les transcriptions des entretiens ont été analysées à l’aide d’une stratégie de codage inductive.

**RÉSULTATS:**

La volonté politique – qu’elle soit nationale ou infranationale – a permis la mise en œuvre de mesures de prévention généralisées au cours de la riposte au COVID-19 dans chaque pays et a stimulé les innovations en matière de prestation de services mobiles et de télésanté. Les participants des trois pays ont souligné l’importance de la mobilisation et de l’engagement des communautés dans les réponses de santé publique et ont noté que l’éducation et l’information sanitaires limitées constituaient des obstacles à la mise en œuvre des efforts de lutte contre la TB au niveau communautaire.

**CONCLUSIONS:**

La volonté politique et la mobilisation sociale doivent occuper une place plus centrale dans les programmes de lutte contre la TB. La conférence COVID-19 a montré que c’était possible. Un niveau d'investissement et de collaboration similaire, voire supérieur, à celui observé lors de la pandémie de COVID-19 est nécessaire pour lutter contre la TB par le biais de partenariats multisectoriels.

The global COVID-19 pandemic and response have reversed many of the hard-won gains made in national TB programmes (NTPs) over the last two decades.^1^ Stringent public health restrictions, health service disruptions and closures, and repurposing of scarce health and human resources to support the COVID-19 response are reported to have led to the downward turn in global TB control efforts.^1^–^3^ Although partial recovery is evident in some high TB burden countries, modelling estimates project that the negative impact will be lasting.^1^,^4^,^5^

Brazil, India and South Africa are among the top 30 high TB burden countries globally with a high estimated number of incident TB cases in the general population; India and South Africa also face concomitant epidemics of drug-resistant and HIV-associated TB. Each country experienced high rates of SARS-CoV-2 infection and mortality throughout the COVID-19 pandemic.^1^ Similar to global trends, their respective NTPs showed a significant and sustained drop in TB case notification, and a consistent rise in the number of TB deaths during the COVID-19 pandemic.^1^ In each country, the COVID-19 response was complex and varied, informed by distinctive political, economic, social and health systems contexts.^6^–^10^

During the first months of the COVID-19 pandemic, the WHO released recommendations for supporting TB services.^11^ Numerous commentaries from public health specialists and TB advocates argue that the unprecedented COVID-19 response in Brazil, India and South Africa – each representative of a distinct global context – holds lessons to inform future TB programmes and services.^8^,^12^–^14^ However, there is limited published research that qualitatively explores these responses and what was feasible in each country. We aimed to explore these lessons from the perspective of TB programme stakeholders in each country, including health service staff and managers, using a qualitative approach, and to make recommendations to support the recovery and robustness of NTPs going forward.

## METHODS

### Study design and setting

This was an exploratory qualitative study involving in-depth interviews with TB stakeholders in selected settings in Brazil, India and South Africa (Table 1).

**TABLE 1:** Description of country health setting and context

	Brazil	India	South Africa
2021 population^1^	214 million	1,410 billion	59 million
2021 TB indicators^1^ Incidence rate Mortality rate	48/100,000 3.8/100,000	210/100,000 36/100,000	513/100,000 93/100,000
COVID-19 indicators (March 2023)^36^			
Total deaths/cases	699,310/37,085,675 (1.9)	530,779/44,690,738 (1.2)	102,595/4,067,067 (2.5)
TB profile^37^	Lower-moderate-endemic setting High estimated number of incident TB cases in the general population and among people living with HIV	Endemic setting High estimated number of incident TB and MDR/RR-TB cases in the general population, and of HIV-associated TB	Severely endemic setting High estimated number of incident TB and MDR/RR-TB cases in the general population, and of HIV-associated TB
COVID-19 response*	Federal Government Questioned scientific evidence and global guidance for COVID-19 response; Undermined coordinated national efforts; Actively disseminated fake news. Local (province or municipal) governments variably implemented Mandatory mask use in public spaces; Social distancing regulations, and Closure of non-essential services. No official country data were collected – these were gathered by a consortium of journals	Unprecedented government responses to the COVID-19 pandemic, including Complete nationwide lockdown Closure of schools and all non-essential businesses Restrictions on movement Mandatory mask wearing Social distancing Screening in all public spaces Border closures Mandatory quarantine for people arriving from COVID-19 affected countries These measures were enforced by government and reduced and reinstated in response to COVID-19 infection rates	
Similar country health profiles	Extreme levels of poverty and inequality Precarious social, political and economic conditions High rates of urbanisation and large populations that rely on free and variable public healthcare compromise health service delivery and access to care Healthcare systems confront insufficient financing, shortage of healthcare professionals, inadequate infrastructure and inequitable access to care, particularly for rural and low-income populations		
Similar TB control strategies and challenges	National TB control programmes primarily implemented at primary care level Programmes are aimed at reducing TB mortality, morbidity and transmission, including Systematic TB screening in communities and health facilities Tracing household contacts of index TB cases, especially those at high risk of disease (e.g., people living with HIV and children <5). Individuals at risk of or presenting with TB symptoms are linked to diagnostic testing and/or TB preventive therapy Daily directly observed therapy is recommended, with regular clinical follow-ups to monitor treatment and side effects for the treatment duration In India and South Africa, nutritional and financial support are available to TB-diagnosed patients to support treatment completion While each country’s national TB control programme is comprehensive, they are not fully implemented. There are significant gaps along the TB care cascade with regards to case-finding, diagnosis, linkage to care and retention in care. TB screening, contact tracing and TB preventive therapy are especially poorly implemented		

*See Biehl et al.,^38^ Fonseca et al.,^39^ and Ferrante et al.^40^ for details on Brazil’s COVID-19 response, GRID COVID-19 Study Group^10^ for details on India’s COVID-19 response, and Rogan & Skinner^41^ for details on South Africa’s COVID-19 response.

MDR/RR-TB = multidrug-resistant/rifampicin-resistant TB.

### Data collection and sampling

In each country, two to three researchers with social or health sciences backgrounds conducted interviews with TB managers and health services personnel between February and August 2022, asking participants to reflect on the COVID-19 response in their countries over time.

In Brazil, we recruited participants from the five Brazilian regions, prioritising high TB burden cities. We sought reference TB services and, after mapping local focal points, used snowball sampling to include ∼5 professionals from primary healthcare and reference centres per region. In India, we collaborated with the State TB Department and purposively selected five primary healthcare centres and one hospital in one Indian city (Chandigarh), ensuring inclusion of facilities with both high and low TB caseloads. We used convenience sampling to identify and invite participants into the study. In South Africa, we collaborated with the Department of Health in two of the country’s high TB burden provinces (Western Cape and KwaZulu-Natal), inviting stakeholders involved in the TB programme at provincial and district levels. To recruit facility-level participants, we purposively selected 12 primary healthcare facilities across the two provinces (ensuring a mix of high and low TB caseloads), inviting ∼3 patient-facing TB personnel at each facility.

Researchers were trained on the study aims and objectives and the study-specific discussion guide. The discussion guide was organised into topic areas with prompts about participants’ experiences of the COVID-19 and TB pandemics and responses, their views of challenges within the health service settings and communities they serve, and lessons for supporting the recovery and robustness of NTPs. Interviews were either online, telephonic or face-to-face to accommodate participant preferences, pragmatic logistical considerations and COVID-19 risk mitigation. Interviews were conducted in the local languages and were 30–90 minutes long.

### Analysis

Interview data were transcribed within-country and translated into English as needed. Six of the authors analysed these data using an inductive coding strategy responsive to the stated aim and objectives, looking for instances where participants spoke about impact, opportunities and lessons. We tabled data by country according to emerging themes and workshopped the themes multiple times to iteratively achieve consensus and identify universal and country-specific experiences and lessons. This process allowed us to triangulate findings across settings. We report our qualitative research following guidelines from the Standards for Reporting Qualitative Research (SRQR).^15^

### Ethics statement

The project was approved by the following research ethics committees: Institute of Social Medicine of the State University of Rio de Janeiro, Rio de Janeiro, RJ, Brazil; All India Institute of Medical Sciences, New Delhi, India; and Stellenbosch University, Cape Town, South Africa. All participants provided informed consent.

## RESULTS

We conducted *n* = 76 interviews across Brazil, India and South Africa (Table 2). We identified two themes which informed lessons for TB programmes and services. The first theme focused on challenges and limitations of delivering TB programmes and services that already existed before the pandemic (sub-theme 1.1) and which were exacerbated by the COVID-19 pandemic, and the related responses (sub-theme 1.2). Participants also shared changes made to TB services to mitigate the impact of the COVID-19 pandemic and response (sub-theme 1.3). The second theme focused on opportunities and lessons from the COVID-19 response to take forward into TB programming, with these opportunities and lessons ­resulting from service delivery innovations for COVID-19. These included leveraging technology (sub-theme 2.1), collaboration and engagement (sub-theme 2.2), mask wearing (sub-theme 2.3), political will (sub-theme 2.4), and considering challenges specific to TB (sub-theme 2.5). The themes and sub-themes are presented in the Figure, with Supplementary Table S1 presenting illustrative quotes under these themes.

**FIGURE:**
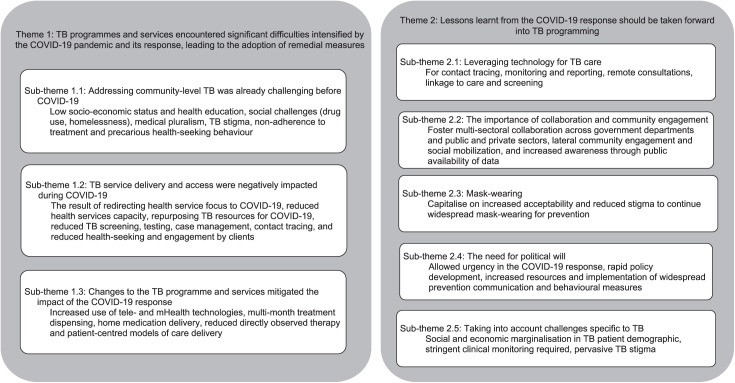
Themes and sub-themes identified using inductive analysis.

**TABLE 2:** Participant types by country

Participant type	Brazil (*n* = 22)	India (*n* = 18)	South Africa (*n* = 36)
Managers	10	5	11
Patient-facing facility staff (or healthcare workers)/	6	9	25
Representatives of community/civil society groups*	6	4	n/a

*Community/civil society engagement reflected the differing contexts in each country. In Brazil where there is strong civil society representation, representatives were activists and national advocacy leaders in TB non-government organisations. In India, representatives were community-based health workers or volunteers who administered directly observed treatment. In South Africa, community/civil society representatives were not included as research participants, although we presented the project and findings to a Community Advisory Board at the affiliated TB research centre.

### Theme 1: TB programmes and services faced substantial challenges exacerbated by the COVID-19 pandemic and response and implemented mitigating changes

#### Sub-theme 1.1: Addressing community-level TB was already challenging before COVID-19

Participants described that TB-affected communities were characterised by low socio-economic status, precarious living conditions, and social challenges (such as drug use, homelessness and TB stigma), which compromised health-seeking behaviour and health services delivery.

Here mostly poor people live and most of them are afraid of going to hospital because they earn and eat daily. (Health worker 9, India)

People here…have less information about TB, even though it’s affecting them the most. They start treatment, and when they start getting better, they stop… You just accept them when they come back, or they’ll disappear again. (Health worker 1, South Africa)

TB is a disease hidden under the rug. People don’t say they have TB, they are ashamed because it is still stigmatised. (Health worker 4, Brazil)

#### Sub-theme 1.2: TB service delivery and access were negatively impacted during COVID-19

In an already precarious TB care context, participants shared how the COVID-19 pandemic and response severely affected TB control efforts, including through prolonged repurposing of scarce TB resources to the COVID-19 response, and through poor case management, patient follow-up and contact tracing. Changes to livelihoods under COVID-19, the result of reduced economic opportunities and informal work, hampered care-seeking and engagement. Limited face-to-face contact between health workers and patients translated into reduced emotional and psychological support for people managed as outpatients.

We had…reduced human [and financial] resources within the TB services because they were shifted to support the COVID-19 response. (Civil society stakeholder 3, Brazil)

All efforts focused on COVID testing…Patients did not come [to the services] and even if they came for [TB] screening, where would their sputum tests be done? (Health worker 7, India)

For social distancing, services were delivered at certain times of the day, and patients had to wait outside, sometimes in bad weather conditions. (Health worker 1, South Africa)

We had limited communication with the patient. If they are facing any issues regarding side effects of medicine, then patients are not coming to hospital because of mandatory COVID-19 testing for all patients. (Health worker 6, India)

#### Sub-theme 1.3: Changes to the TB programme and services mitigated the impact of COVID-19

Participants also described changes to routine TB service delivery to mitigate the impacts of COVID-19. These included increased use of digital health technologies for consultations with patients, multi-month medication dispensing, home medication delivery and reduced directly observed therapy (DOT) in the first 2 weeks of treatment. Some participants who were managers described these changes as representing more patient-centred TB care delivery.

Lockdown was imposed by the government…so we started telemedicine services. (Health worker 8, India)

We tried to do HIV-style [medication dispensing for TB] by dispensing more than 30 days [of medication]. Where there was a structure … to take the medicine to people’s homes … We tried these strategies to avoid crowding in health centres. (Manager 14, Brazil)

Even pre-COVID, I always thought… [we should] put …more trust in patients and their families, and treatment supporting at home, rather than asking someone to come daily to the clinic to watch them swallow a tablet. (Manager 5, South Africa)

### Theme 2: Lessons learnt from the COVID-19 response should be taken forward into TB programming

In all three countries, participants described opportunities opened and lessons learnt by the COVID-19 responses that they believed held promise for supporting their TB programmes going forward. These included leveraging technology to support the TB programme and services, increasing multi-sectoral collaboration and community engagement, leveraging more acceptable mask wearing, and garnering political will for TB.

#### Sub-theme 2.1: Leveraging technology for TB care

Innovative use of technology in the COVID-19 response could similarly be used for TB, with numerous examples of mobile health (mHealth) applications and tools developed. In Brazil and India, virtual consultations with people undergoing TB treatment were especially useful to ensure continuous care and emotional support and served as a replacement for facility-based DOT. In South Africa, the COVID-19 response resulted in a provincial public-facing TB data dashboard, SMS-based notification of negative TB results to patients, a mHealth application for TB self-screening and initiatives to use routine health data to triage and support TB patients at high risk of mortality.

They had digital apps [for COVID-19] and the information could flow…you could know what changed yesterday… You would know the index case had twenty contacts … they’ve scanned only five … Contact management for TB is still paper-based. (Manager 10, South Africa)

Just like the *Aarogya Setu *app for COVID-19, there should be a similar app [for TB] … the patient can make a video call to me [and I can see] every day [he] has taken medicine. (Health worker 1, India)

#### Sub-theme 2.2: The importance of collaboration and community engagement

Participants in India and South Africa, where there was a strong government response, emphasised that multi-sectoral collaboration was critical to their country’s comprehensive COVID-19 responses. Collaborations and engagements with sectors outside of health allowed massive targeted public health messaging across multiple media platforms; broader skillsets could be leveraged in the response, increasing urgency and reach. Engagement with many different actors and their interests facilitated more lateral and transparent relationships between Ministries of Health, communities and other partners. In Brazil, participants shared how the federal government’s COVID-19 denialism resulted in the response being driven by local governments and civil society. Despite the central government’s tenuous support, alignment of medical and scientific communities enabled implementation of various prevention measures and virtual support, and monitoring and surveillance systems. Participants in all three countries emphasised the importance of mobilising and engaging communities in public health responses, and including traditional practitioners in India and South Africa. ­Similarly, participants noted limited health education and information, and hierarchical relationships as barriers to implementing TB control efforts at the community level. In all three countries, public-facing data dashboards helped spread awareness of how the COVID-19 pandemic and response were progressing and were thought to encourage preventive health behaviours.

We used the multi-sectoral approach [and] did multi-faceted publicity; social media, TV, newspaper, pamphlets, posters and banners of COVID were pasted onto rickshaws and buses … We did everything to reach every nook and corner. (Manager 4, India)

We’ve learnt that … if community leaders aren’t with you from the start… they become the biggest barrier at a community level; the trust that communities have in them is so much more than they have in us. (Manager 13, South Africa)

#### Sub-theme 2.3: Mask-wearing

Participants across the three countries shared that mask-wearing had become less stigmatised as a result of the COVID-19 ­pandemic. They emphasised how increased acceptability of mask-wearing was an opportunity for TB; people had become more aware of the protective effects of masks and that people wear masks for a variety of reasons, reducing the association with TB.

[Mask-wearing] became widespread and popular…This was a gain in that it made the population aware of using this resource in a … less stigmatised way. (Health worker 22, Brazil)

#### Sub-theme 2.4: The need for political will

In India and South Africa, participants saw the COVID-19 response highlight the comparative complacency and tenuous political prioritisation of the TB programme. The COVID-19 responses in these two countries showed how political will allowed urgency in the response, enabled multi-sectoral and community engagement and opened up access to resources. In all three countries, participants explained how political leadership facilitated widespread prevention communication and behavioural measures to be disseminated rapidly.

The urgency is because we had the Health Department and the political principals pulling in the same way…In TB, we don’t get that political support. (Manager 10, South Africa)

Official channels…took a long time to manifest/respond about the pandemic. We have a government with inconsistent messages, a sector saying to do one thing and …another advising to do another. (Civil society stakeholder 3, Brazil)

#### Sub-theme 2.5: Taking into account challenges specific to TB

Some participants also noted challenges related to differences between TB and COVID-19. TB-affected populations were perceived to experience greater social and economic marginalisation than the general population, and to have lower health literacy and education, in addition to experiencing TB stigma. They described TB treatment as complex, requiring regular monitoring for treatment response and side effects over a relatively short treatment period, while COVID-19 involved largely behavioural and vaccination-driven interventions. Some participants cautioned against service delivery innovations that would decrease interactions between providers and patients. In South Africa, participants were hesitant about being able to reach patients by telephone consistently, when there was high turnover of telephone numbers among patients.

## DISCUSSION

The negative impact of the COVID-19 pandemic and response on infectious and non-communicable disease programmes alike are widely reported.^16^,^17^ For TB programmes, many of which reported slow declines in TB incidence and mortality rates even before the COVID-19 pandemic, this has meant the reversal of decades of progress in improving TB case notification, diagnosis and reducing mortality; it has also compounded already sub-optimal progress towards achieving the targets of the WHO End TB Strategy.^18^ The similarities between COVID-19 and TB – both airborne diseases involving similar behavioural methods for prevention and control – have subsequently led TB policy makers, researchers and programme implementers to draw comparisons between COVID-19 and TB responses in search of lessons to support the recovery of NTPs and improve their delivery going forward.^8^,^13^,^14^,^19^–^24^ In our study, we qualitatively explored opportunities and lessons from the COVID-19 responses of three high TB burden countries – Brazil, India and South Africa – to inform implementation of more robust TB programming and services going forward. We found that participants saw value in many of the actions and innovations that were implemented and developed in the COVID-19 response for the TB programme. To variable degrees across the three countries, these included the use of digital and mHealth technologies for virtual consultations, screening, contact tracing, and monitoring and reporting, multi-sectoral involvement, community engagement and mask-wearing. In India and South Africa, many participants lauded the governmental political will and urgency that accompanied the COVID-19 response, believing it to be crucial as a catalyst and enabler of the response. In Brazil, the absence of support from the federal government had the COVID-19 response be variably led by local governments and civil society. Although each country in this study exhibits unique TB epidemiology and responds differently to COVID-19, the essential components for effective programming are evident in all three settings. Arguably, limitations in each country’s response both for TB and COVID-19 are suggestive of not leveraging all of these ingredients simultaneously or optimally. For example, South Africa has had massive investment into expanding HIV and TB responses over time, concomitantly strengthening its health system. These investments allowed the infrastructure and systems necessary to mount a robust COVID-19 response, even as they have continued to be sub-optimally implemented for TB. Our findings add to a growing body of literature recognising political will, multi-sectoral engagement and community-led participation as crucial to recover and maximise TB programme delivery.^8^,^14^,^24^–^26^

Our study has several strengths. Qualitative data from three high TB burden countries which experienced high rates of SARS-CoV-2 infection were collected and analysed to identify lessons for improving future TB programming. Purposive selection of key informants from diverse health system cadres across the three countries adds to the strength of the design. The similarities that we found in the data across the three distinct country contexts add to the strength of our findings. Extrapolating lessons and opportunities from the COVID-19 responses should be limited to places where underlying disease and health service dynamics are similar.

Globally, we are in a critical moment for TB to leverage the increased global attention to the setbacks in TB elimination efforts caused by the COVID-19 pandemic and responses and to transfer the lessons and opportunities for creating more robust TB programmes and services. For too long, political will for TB has been tenuous. This is largely attributed to the fact that as the disease burden is primarily concentrated in low- and middle-income countries, investment in TB research and development has been decreasing when compared to other well-established and emerging infectious diseases like HIV.^25^,^27^–^31^ Investment into and prioritisation of TB as a key area of global health is vital to enable affected governments – who often suffer already weak and inefficient health infrastructure and systems – to align with and mobilise towards achieving global End TB targets.^31^

The unparalleled public health communication efforts during the COVID-19 response, along with the heightened awareness they generated in the general population regarding infection prevention, control measures and behaviour to reduce transmission, established a crucial groundwork for engaging the public in addressing the TB burden and its control, as well as for mobilising political support. As in our study, others similarly highlight the potential for integrated digital data systems alongside public access to real-time TB data to create awareness of the worsening TB epidemic and to inspire community-led advocacy and pressure to scale up TB responses and funding, and call for improved TB diagnostics and treatments.^8^,^13^,^14^ This could also help to promote continued mask-wearing, which is widely reported as having become a less stigmatised practice following the COVID-19 pandemic.^32^ Many studies also report the value of digital and mHealth technologies to support and supplement clinical management of TB at home and virtually, as well as service adaptations to offer more flexible access to medications in places of the patient’s choosing.^8^,^25^,^33^ Such innovations in TB service delivery are long overdue as they have been thought to go against the interests of TB control. Implementing such adaptations has the potential to move TB towards a more person-centred service.^34^,^35^ Importantly, any of these actions alone will not recover the TB programme. Zimmer et al. argue for adoption of a ‘Swiss Cheese Model for Ending TB’ which emphasises the compounding effect of multiple interventions aimed simultaneously at societal, personal and health system levels,^24^ and includes the opportunities and lessons highlighted in this paper. As with COVID-19, a holistic response is needed for TB – often already articulated in global and national plans, but poorly implemented without concomitant political will and financial investment. Our participants’ experiences show that one of the most important lessons from COVID-19 is that we can, should and must, do more and better for TB.
