# Supplementary material for: Lessons for TB from the COVID-19 response: qualitative data from Brazil, India and South Africa
Source: Public Health Action. 2023 Dec 7;13(4):162–8. doi: 10.5588/pha.23.0044 (PMC10703135; doi:10.5588/pha.23.0044)
Supplement: Supplementary file 1 [file iutld_pha_23.0044_supplementarydata1.pdf]

http://dx.doi.org/10.5588/pha.23.0044

Lessons for TB from the COVID-19 response: qualitative data from Brazil, India and South Africa

Supplementary Table S1. Illustrative quotes from interviews with key informants

**Theme 1: TB programmes and services encountered significant difficulties intensified by the COVID-19 pandemic and its response, leading to the adoption of remedial measures**	
**Sub-theme 1.1: Addressing community-level TB was already challenging before the COVID-19 pandemic**	**Illustrative quotes**
Low socio-economic status<br><br>Social challenges<br><br>Health education<br><br>Health-seeking behaviour<br><br>Non-adherence<br><br>Medical pluralism<br><br>TB stigma	“Here mostly poor people live and most of them are afraid of going to hospital because they earn and eat daily. The hospital is overcrowded and if a person goes there his whole day gets wasted.” Health worker 9, India<br><br>“This community, you would think that most people are literate now and understand [health] stuff, but people here…have less information about TB, even though it’s affecting them the most. They start treatment, and when they start getting better, they stop… They’re just not getting it. You just accept them when they come back, or they’ll disappear again.” – Health worker 1, South Africa<br><br>“The community health teams are in the communities giving health education…but we still have this problem of TB… We [need to] target the traditional health healers in the community because honestly, if our patients … feel sick, the first people that they will consult is traditional healers.” Manager 19, South Africa<br><br>“The patient does not want to tell their families [about their TB diagnosis] so they make it clear to us that…they will throw them from the home. ‘Where will I stay? And if I will be expelled from my job, how I will earn?’” Health worker 5, India<br><br>“With TB they share [the diagnosis] with family members quite easily because they live around people who have TB. They know they can come to the clinic…and you feel better…But I feel like people are desensitized to it. They only seek medical help or treatment when they are unwell, or it affects their activities of daily living. And after that it’s like they quickly forget where they were, and they default and they end up in the same place again.” Health worker 1, South Africa<br><br>“I think we could have more social engagement [in TB], which we don't have…TB is a disease hidden under the rug. People don't say they have TB, they are ashamed because it is still stigmatised.” Health worker 4, Brazil

	“We have two main aspects: that of patients who have been very impoverished, many people have lost their jobs, a source of income. Many people are relying on social aid ... going through financial difficulties ranging from losing their health insurance to losing their homes.” Health worker 19, Brazil
**Sub-theme 1.2: TB service delivery and access were negatively impacted during COVID-19**	**Illustrative quotes**
Focus on COVID-19 response<br><br>Reduced health services capacity<br><br>TB resources repurposed for COVID-19<br><br>Reduced TB screening, testing, case management, contact tracing<br><br>Reduced health-seeking and engagement by clients	“We had two major problems [in TB], reduced human resources within the TB services because they were shifted to support the COVID-19 response, and reduced financial resources that ended up also being used in most of the emergency actions for COVID-19.” Civil society stakeholder 3, Brazil<br><br>“[TB] screening was not even done, all efforts focused on COVID testing, so it was not even possible to conduct TB tests, because where would the patient go? Patients did not come [to the services] and even if they came for screening, where would their sputum tests be done? There have been a lot of problems [in microscopy], the patients’ [TB] tests were not done for many days.” Health worker 7, India<br><br>“[Potential] new [TB] patients – those who had cough and cold – they were not coming [to the hospital] because of fear of getting COVID positive. At that time patients had also started coming [to the hospitals] less and some doctors started spending more time in COVID, it [TB case notification] decreased … because all the staff started working for COVID-19.” Health worker 13, India<br><br>“Facilities operated on a reduced capacity – only a certain number of patients were allowed in the facility at a time for social distancing, services were delivered at certain times of the day, and patients had to wait outside, sometimes in bad weather conditions.” Health worker 1, South Africa<br><br>“A lot of imaging tests were requested during COVID, and I think that imaging tests are very important for TB. The moment you have more access to x-rays, you also end up having a greater chance of diagnosing a disease through imaging. We had this resource for TB and it was underused.” Health worker 22, Brazil<br><br>“One patient shared with us that ‘I have already faced the baton of police so I will not come to get medicine.’” Health worker 2, India<br><br>“Many people have lost their jobs…and found themselves unable to access health services…And the number of homeless people that we have been assisting is growing, this makes any activity in the health area more difficult.”

	Health worker 19, Brazil<br><br>“We just had limited communication with the patient. If they are facing any issues regarding side-effects of medicine, then patients are not coming to hospital because of mandatory COVID-19 testing for all patients.” Health worker 6, India
**Sub-theme 1.3: Changes to the TB programme and service mitigated the impact of the COVID-19 response**	**Illustrative quotes**
Increased use of tele- and mHealth technologies<br><br>Multi-month dispensing<br><br>Home medication delivery<br><br>Reduced DOTs<br><br>Patient-centred care	Lockdown was imposed by the government…so we started telemedicine services…It didn’t just happen in our Centre but happened all over India.” Health worker 8, India<br><br>“We video called the patients and tell patients to eat medicine in front of us…For those who didn’t have smartphones, we used to visit them.” Health worker 12, India<br><br>“We tried to do HIV-style [medication dispensing for TB] by dispensing more than 30 days [of medication]…Where there was a structure … to take the medicine to people's homes so they wouldn't go to the health unit, we tried a lot of these strategies to avoid crowding in health centres.” Manager 14, Brazil<br><br>“[We took] some actions in relation to DOT [Directly Observed Treatment]. Some patients made a video, taking their medication, so there were some strategies used during this period... Many people saw it as a positive experience because they were able to monitor the health system user and maintain some bond with that person within what was possible at that time” Health worker 12, Brazil<br><br>“There was a diversion of human resources to work with COVID-19, closure of basic health units and changes in consultations, observed treatment... We guided some successful experiences that could contribute to reducing the loss of cases as much as possible. So some actions, at that time, were to use the phone and WhatsApp a lot to monitor these cases… So this was the most used technology.” Manager 6, Brazil<br><br>“We started doing home deliveries [for HIV, TB, and chronic disease]. Even using Uber drivers to deliver medication. There was lots of innovations at the time: home deliveries, using the NPOs (non-profit organisations), having [medication] pickup points in the community. Patients were still not coming [to facilities] after a while, because they were still scared of being infected. The main thing from the facility [was that] we could give [patients] a month’s supply [of treatment], two months, we did that as well.” Manager 5, South Africa

	"I gave one week's medicine in advance to the patient…[and] called one family member…along with the patient and told them that I am giving this medicine to you and it's their responsibility now to ensure that the patient is having medicine on time. After one week [they would] come to me with empty medicine strips." Health worker 9, India<br><br>"Even pre-COVID, I always thought, do you really need someone coming to the clinic every day [for DOTs]. It's almost, like the policing thing. [We should] put a bit more trust in patients and their families, and treatment supporting at home, rather than asking someone to come daily to the clinic to watch them swallow a tablet." Manager 5, South Africa
**Theme 2: Lessons learnt from the COVID-19 response should be taken forward into TB programming**	
**Sub-theme 2.1: Leveraging technology for TB care**	**Illustrative quotes**
Contact tracing<br><br>Monitoring and reporting<br><br>Remote consultations<br><br>Linkage to care<br><br>Screening	"They had digital apps [for COVID-19] and the information could flow…you could know what changed yesterday… You would know the index case had twenty contacts, so far, they've scanned only five. Whereas the contact management for TB is still paper based." Manager 10, South Africa<br><br>"The COVID-19 pandemic has led to the introduction of new technologies…such as apps to help monitor high-incidence areas, prevent transmission... This could be used for TB as well." Manager 12, Brazil<br><br>"After the pandemic… there were some increases in the quality of the service that we provide with regard to tuberculosis, especially with regard to remote contact. We used the telephone a lot to talk to our TB patients…and we started to do the treatment directly observed by video on WhatsApp… We learned with COVID that we could do it and it worked." Health worker 19, Brazil<br><br>"Just like the AarogyaSetu app for COVID-19, there should be a similar app [for TB]. Many times [TB] patients cannot come, then there should have been a system through which patients can get online reports…The patient can make a video call to me [and I can see] every day [he] has taken medicine." Health worker 1, India<br><br>"If we had a system like we had for COVID where [patients] get an SMS with a link and they go to the closest facility. They will be able to follow the link and add your results right there and commence treatment with you, [that would support continuity between urban and rural areas]." Manager 11, South Africa

	"[The COVID-19 screening app] is very user friendly. It has your questions for COVID screening. I think asking those questions might take, maybe two, three minutes to actually fill that on the app. And then it classifies your patients as someone who needs to be tested, and you also record that you are testing the patients. And it has a prompter. It will also call back the result, then patients get their results via SMS. By the time the health worker knows, the patient also knows." Manager 10, South Africa
**Sub-theme 2.2: The importance of collaboration and community engagement**	**Illustrative quotes**
Multi-sectoral collaboration<br><br>Community engagement/social mobilization<br><br>Public data dashboards	"We used the multi-sectoral approach and worked in 360 degrees; we did multi-faceted publicity. We used multimedia like social media, TV, newspaper, pamphlets, posters, and banners of COVID were pasted onto rickshaws and buses. We even use mics [microphones] and informed people to get themselves tested, maintain social distance and wear masks, we did everything to reach every nook and corner." Manager 4, India<br><br>"We've learnt to further explore [communication] networks for tuberculosis. [We used] advertising on the [public] buses… it was used a lot in the pandemic to bring information about COVID. There are some learnings in the ways of disseminating information in the media, not the material necessarily, but the mediums used. It is important for us to explore [this for TB] because the population tends to look at these places a lot." Health worker 9, Brazil<br><br>"The [government] and the NPOs (non-profit organisations) and the academics came together to mount a response against this pandemic. Never seen before, that we use everyone's skills." Manager 5, South Africa<br><br>"In the past the Department of Health … approached [health interventions] from a position of superiority, you know, 'we are the authority on this topic, and we will tell you how to do it.' And through COVID we've learnt that that doesn't work with communities, they need to be part of the process in terms of how you go about implementing new programmes… If these community leaders aren't with you from the start… they become the biggest barrier at a community level because their influence and reach within the communities is so much and the trust that communities have in them is so much more than what they have in us." Manager 13, South Africa<br><br>"What we did as a civil society, we communicated and guided people who had respiratory diseases, asthma, tuberculosis, to look out for the signs and seeking care as needed. We achieved this through activism." Civil society stakeholder 18, Brazil

	“It would benefit the community [to have] the [TB] data out there because they will see the seriousness of the disease, of how the disease is quickly spreading. Same as for COVID, the data was out there. They could see that this is spreading quickly, so they need to act swiftly…go into the facilities to be investigated for TB.” Manager 19, South Africa.
**Sub-theme 2.3: Mask-wearing**	**Illustrative quotes**
Increased acceptability<br><br>Reduced stigma	“I think we had a gain in the sense of using masks, it was something that became very widespread and popular. People now no longer have this… fear of wearing a mask …This was a gain in the sense of making the population aware of using this resource in a … less stigmatized way.” Health worker 22, Brazil<br><br>“For TB [the mask] was the best thing in the sense of stigma. Before, if you had to use a mask, it would be pointed out that the person was sick. Now it was the opposite. Not only those who had COVID needed to wear a mask to protect others, but the mask was much more for everyone's protection. So I believe that the use of the mask has become normalised and…anyone who wants to wear a mask will use it.” Health worker 9, Brazil<br><br>“Wearing a mask was stigma, if I will wear it then everyone will get to know that I am a TB patient, but now you can see many people wearing a mask so the stigma regarding mask wearing has been reduced, people are now wearing it for their own safety and prevention.” Manager 3, India<br><br>“[Mask wearing] is one plus for the TB programme. It’s becoming a bit more acceptable in the communities, in the clinics, so that’s positive. There’s still a massive stigma for TB and DR-TB but I think [mask wearing has] helped a lot. You will stand out more if you don’t wear a mask.”<br>Manager 5, South Africa
**Sub-theme 2.4: The need for political will**	**Illustrative quotes**
Created urgency<br><br>Rapid policy development<br><br>Increased resources and rigour	“I think the urgency is because we had the Health Department and the political principals pulling in the same way. The Health Departments do what the policy makers tell it to do. You know if the Minister of Health says jump, you jump. Whereas in TB we don't get that political support for whatever we're trying to do. With COVID … you certainly had strong political commitment.” Manager 10, South Africa<br><br>“We saw more COVID policies of higher quality coming out of the provincial office in the space of a month, than we saw coming out over years before in all the programme areas…There was an unprecedented collaboration

	and involvement of public health specialists in the policy process, which before had been in programme specific silos. It just brought a whole other level of quality. And then I think with the urgency [around COVID-19]." Manager 7, South Africa<br><br>"For COVID publicity, separate funds were provided by the government of India, there was a separate budget for everything, and we have been given guidelines from the government." Manager 4, India<br><br>"Like in COVID-19 time if a person is positive in an area then all people in his area or who live nearby him were traced; wherever he went or travelled, tests were done on his contacts, and if they were found positive, then they were also isolated or all the houses in his building were tested. So, the same thing could be happened in TB as well." Health worker 5, India<br><br>"I think we made a lot of mistakes [as a country]. We had official channels, such as the Ministry of Health, which took a long time to manifest/respond about the pandemic. We have a government with inconsistent messages, a sector saying to do one thing and then another sector advising to do another... There were considerations completely contrary to what science had been saying." Civil society stakeholder 3, Brazil<br><br>"[We] saw four, five thousand deaths a day. And especially in the beginning [of the pandemic] when we didn't know how to treat patients. We were not enough professionals. We had to learn... Then the vaccination begins, and you have more fake news. With the political difficulty that we had from a government that did not support science at any time. And in fact, we know very well about the hate cabinet and the question we had regarding the use of chloroquine hydroxide... and even today we have fake news related to COVID." Health worker 4, Brazil
**Sub-theme 2.5: Taking into account challenges specific to TB**	**Illustrative quotes**
TB patient demographic<br><br>Complicated clinical TB care<br><br>TB stigma	"The patient profile that we had with COVID is a little bit different from what we have with our TB patients. A lot of the [COVID] patients that we phoned were actually…private patients, younger patients, and they were not very sick in most cases…Whereas with TB patients they tend to be much sicker…And then there's complications with their comorbidities and HIV and other things that they might have. And then also, you have to take some time to counsel them on their clinical condition before you get to talking about contacts and other things. So, it's a bit different. With COVID it was fairly straightforward in most cases." Manager 6, South Africa<br><br>"Some of the difficulties with telehealth [is that] you are reliant on people having access to a phone and a working phone. TB patients are more likely to be of a demographic that that's not as prevalent, that kind of

	[telephone] access.” Manager 7, South Africa<br><br>“Access to phones and data is variable. We have a very large segment of people that only have WhatsApp. They are not people who would be able to chat on video as we are. And even WhatsApp access is erratic. Because even their account doesn't have free mobile data. So he has to go somewhere to turn on the Wi-Fi so he can talk to us. And we have this very big barrier. We have a reasonable number of people who don't have cell phones or who don't have any communication access.” Health worker 19, Brazil<br><br>“I think the stigma of COVID doesn't exist basically because people who get infected by COVID feel normal for having COVID. Everyone can have it. But TB is still associated with an economically disadvantaged population, a more marginalized population. So there is prejudice with the diagnosis.” Health worker 22, Brazil
